# Ileal Transcriptome Profiles of Japanese Quail Divergent in Phosphorus Utilization

**DOI:** 10.3390/ijms21082762

**Published:** 2020-04-16

**Authors:** Michael Oster, Henry Reyer, Nares Trakooljul, Frank M. Weber, Lu Xi, Eduard Muráni, Siriluck Ponsuksili, Markus Rodehutscord, Jörn Bennewitz, Klaus Wimmers

**Affiliations:** 1 Leibniz Institute for Farm Animal Biology (FBN), 18196 Dummerstorf, Germany; oster@fbn-dummerstorf.de (M.O.); reyer@fbn-dummerstorf.de (H.R.); trakooljul@fbn-dummerstorf.de (N.T.); weber@fbn-dummerstorf.de (F.M.W.); xi@fbn-dummerstorf.de (L.X.); murani@fbn-dummerstorf.de (E.M.); ponsuksili@fbn-dummerstorf.de (S.P.); 2 Institute of Animal Science, University of Hohenheim, 70599 Stuttgart, Germany; markus.rodehutscord@uni-hohenheim.de (M.R.); j.bennewitz@uni-hohenheim.de (J.B.); 3 Faculty of Agricultural and Environmental Sciences, University Rostock, 18059 Rostock, Germany

**Keywords:** phosphorus use, intestinal expression profiles, fowl, quail physiology

## Abstract

Phosphorus (P) is an essential component for all living beings. Low P diets prompt phenotypic and molecular adaptations to maintain P homeostasis and increase P utilization (PU). Knowledge of the molecular mechanisms of PU is needed to enable targeted approaches to improve PU efficiency and thus lower P excretion in animal husbandry. In a previous population study, Japanese quail were subjected to a low P diet lacking mineral P and exogenous phytase. Individual PU was determined based on total P intake and excretion. A subset of 20 extreme siblings discordant for PU was selected to retrieve gene expression patterns of ileum (*n* = 10 per PU group). Sequencing reads have been successfully mapped to the current *Coturnix japonica* reference genome with an average mapping rate of 86%. In total, 640 genes were found to be differentially abundant between the low and high PU groups (false discovery rate ≤ 0.05). Transcriptional patterns suggest a link between improved PU and mitochondrial energy metabolism, accelerated cell proliferation of enterocytes, and gut integrity. In assessing indicators of the efficient use of macro- and micronutrients, further research on turnover and proliferation rates of intestinal cells could provide an approach to improve P efficiency in poultry species.

## 1. Introduction

Phosphorus (P) is essential for all living beings as it has a key role in many biological processes, including bone mineralization and energy metabolism. The maintenance of P homeostasis is therefore crucial for ensuring the physical integrity of an organism. Due to physiological turn-over and growth processes, the dynamics of P absorption, P storage, and P excretion are of particular importance to maintain animal health and growth performance. The resulting interaction of tissues such as small intestine, bones, and kidneys prompt an individual physiological release of hormones [1], tissue-specific downstream signaling events [2], and modified bone mineralization [3] to account for varying environmental conditions. With respect to P, physiologic adaptations imply effects on gastrointestinal processes, intermediate metabolism, and mechanisms of resource allocation, which all affect the efficiency of P utilization (PU) of the animal.

Due to economic constrains, ecological burden, and finite rock phosphate resources, it is desirable to improve PU in farm animals. Animal-intrinsic factors of the P-homeostasis have been extensively studied—for instance, in Japanese quail (*Coturnix japonica*) with a particular focus on the genetic contribution of PU [4,5]. It is considered a valuable model organism for avian species due to the lower space requirements of the mature birds and their relatively short generation interval of a few weeks [6,7]. Consequently, a large experimental quail population was generated in which individual feed intake and P excretion rates were recorded to approximate individual PU [5]. The genetic analysis of this quail population revealed a low to moderate heritability of PU. Moreover, positive phenotypic correlations between PU and traits such as bone characteristics (i.e., foot and tibia ash) have been described in these quail [4]. These observations are in line with recent results on genetic parameters estimated for traits related to PU including blood levels of P, Ca, and alkaline phosphatase activity in other monogastric species, thus emphasizing the contribution of animal-intrinsic factors on the variability of the PU [8].

The intestinal P absorption is facilitated and quantitatively improved by supplementation of mineral P derived from inorganic P sources such as monosodium phosphate. In monogastrics, the proximal parts of the small intestine are considered the main sites of mineral P uptake. In contrast, plant P mainly originates from phytic acid (inositol hexakisphosphate; InsP_6_) and its salt phytate which represent the principal storage forms of P in plant seeds [9]. The stepwise degradation of dietary InsP_6_ by phytases and other phosphatases results in the gradual release of phosphate and enables the uptake of P and myo-inositol. The capacity to hydrolyze InsP_6_ and its degradation products along the gastrointestinal tract of chicken is considerable [10] but likely dependent on multiple factors including microbial activity, dietary composition and dietary mineral levels as shown recently [11,12]. In gnotobiotic broiler chicken, 42% of dietary InsP_6_ was found hydrolyzed at the terminal ileum when a diet free of phytase was provided [13]. However, extensive studies in monogastric species revealed the occurrence of a considerable amount of polyphosphorylated inositols (e.g., InsP_6_, InsP_5_, InsP_4_) in distal gastrointestinal sections [14] and therefore the potential to make further use of plant-derived P sources. It remains elusive whether functional endogenous phytases and corresponding sodium-phosphate co-transporters are expressed in the respective intestinal mucosa sections, although some studies claimed their existence [15,16,17]. Clearly, intestinal tissues contribute to the inter-individual variation of PU and thus to P efficiency by balancing animal requirements, intrinsic responses, and environmental stimuli.

This study is based on a large quail population which has been extensively phenotyped to estimate individual PU values [5]. Ileum samples of quail exhibiting either high or low PU were subjected to the mRNA sequencing approach to generate gene expression profiles. The identification of genes and molecular pathways related to PU will contribute to understand the intestinal capacity for improved P efficiency in avian species.

## 2. Results and Discussion

Previous trials in monogastric species showed that a lowered supply of dietary P prompts an altered expression of transcellular P transporters in the intestine and kidney [17,18] while lowering renal P excretion [19,20] and bone mineralization [3,20]. Moreover, the vitamin D system has proven to be one of the major molecular signaling cascades for dealing with dietary P restrictions [21]. Investigations on the regulation of P homeostasis revealed initial adaptation processes to divergent dietary P supply at the level of the parathyroid gland via parathyroid hormone (PTH) secretion with known implication on bone, kidney and intestine in pigs and broilers [1,22,23]. PTH, which primarily targets calcium serum levels, also showed short-term and long-term effects on intestinal transporter activity and the proliferation of enterocytes [24,25]. Specifically for poultry, a recent chicken study revealed that a low P diet induced negative effects on feed intake, but improved the ability to synthesize adenosine triphosphate (ATP) in enterocytes [26]. Broiler chicken fed low P diets consistently showed higher phytate-P release and absorption in the intestine compared to conventionally fed chicken [27]. This seems to happen at the expense of body growth performance and bird survival [26]. Obviously, responses to reduced dietary P supply prompt a certain individual variability at the molecular level. Animals classified as high PU are assumed to be capable of achieving appropriate molecular adaptations. In this context, specific gene expression patterns of PU are used to clarify potential transcriptional mechanisms responsible for these adaptations.

### 2.1. Sequencing Data Analysis and Differential Gene Expression

**Figure 1:**
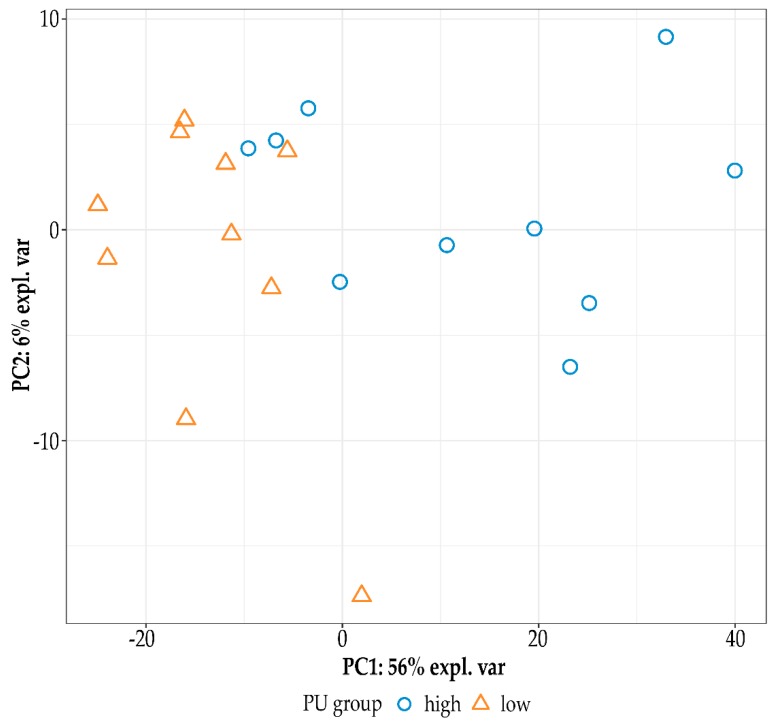
Principal component analysis based on the profiles of differentially expressed genes (q-value ≤ 0.05) in ileum samples of quail exhibiting low (orange triangle) or high (blue circle) phosphorous utilization (PU).

In the present study, 20 libraries from discordant full siblings of quail divergent in PU were analyzed by high throughput mRNA sequencing. The sequencing of all 20 ileum samples revealed a mean number of 24.9 million reads with an average mapping rate against the current *Coturnix japonica* reference genome of 85.6%. This corresponds to a total number of 12,684 unique genes that were used for downstream analysis. 640 of these genes exhibited different levels of expression between the experimental groups (q-value ≤ 0.05). The list of differentially expressed genes (DEGs) contains 342 up-regulated (H > L) and 298 down-regulated (H < L) genes (Supplementary Table S1). Under consideration of the DEGs, quail with low and high PU separate due to the first two principle components in the PCA (Figure 1). Although one animal from the low PU group showed some uncertainty, successful discrimination of PU divergent animals based on ileal gene expression underlines the relevance of the intestinal capacity and the involvement of host genetic factors in the population-wide variability of PU as previously indicated by heritability estimates for this trait [5]. Accordingly, studies in non-avian monogastric species such as pigs revealed a gene-environment interaction in relation to P homeostasis [2,8,17]. Using the ileal DEGs, a functional enrichment analysis was performed to further clarify the underlying mechanisms and the intestinal contribution to PU. For the overall interpretation of the transcriptomic profiles of the current study, it has to be considered that all quail were kept on the same low P diet during the adaptation period and throughout the trial.

### 2.2. Gene Ontology (GO) Analysis

**Figure 2:**
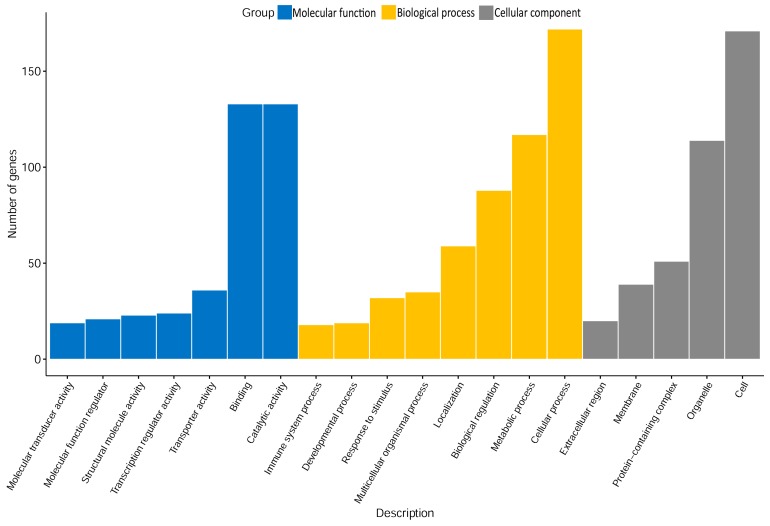
The Gene Ontology (GO) classification considering the 640 genes being differentially expressed between quail assigned to high and low PU groups. Displayed GO terms in the categories molecular function, and biological process and cellular component include at least 10 differentially expressed genes (DEGs).

The identified DEGs were annotated in different Gene Ontology (GO) term categories (Figure 2). With regard to the molecular functions category, a substantial number of genes was annotated in “binding” and “catalytic activity”. For biological processes, the terms “metabolic and cellular processes” were accumulated. In particular, the overrepresentation test implemented in the PANTHER software revealed an enrichment of the DEGs in the more specific terms “mitochondrial ATP synthesis coupled electron transport” and “regulation of cell adhesion” (q-value ≤ 0.05). GO terms related to “organelle” and “cell” were the most abundant in the cellular components category, with an overrepresentation of the specific terms “mitochondria”, “apical plasma membrane”, and “membrane rafts and adherens junctions” (q-value ≤ 0.05). The most abundant protein classes encoded by the identified DEGs were each referred to 8–15% of “nucleic acid binding”, “hydrolase”, “transcription factor”, “enzyme modulator”, “transporter”, and “transferase”. Overall, the GO term analysis revealed two main themes in the comparison between the two groups, which refer to mitochondrial energy metabolism and cell junctions. In order to assess the impact of effects at the molecular level (i.e., activation or inactivation in high PU), a downstream analysis of corresponding canonical pathways was performed.

### 2.3. Biological Pathway Analysis

**Figure 3:**
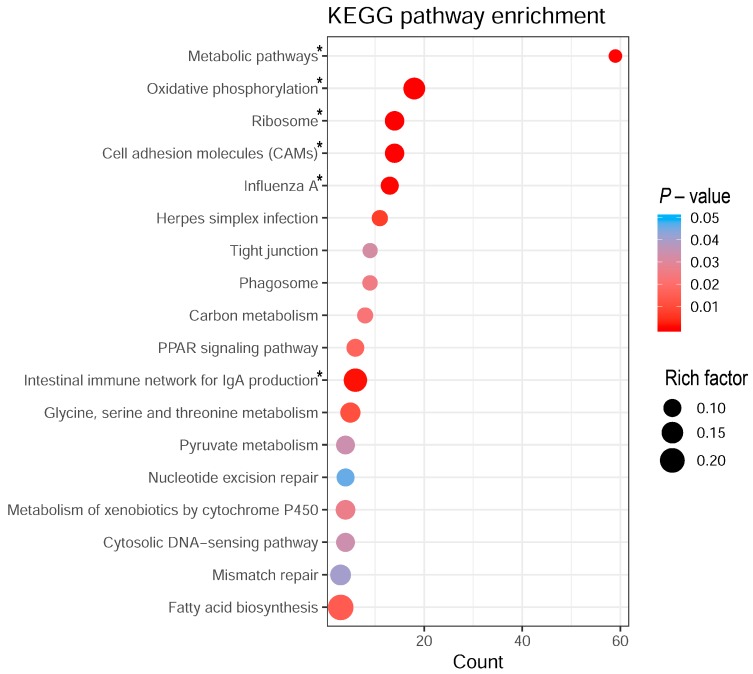
Kyoto Encyclopedia of Genes and Genomes (KEGG) pathways deduced from the 640 differentially expressed genes (DEGs) between quail assigned to high and low PU groups. Significantly enriched pathways are shown (*p*-value ≤ 0.05). Pathways indicated by an asterisk remain after multiple testing correction (Benjamini–Hochberg adjusted *p*-value ≤ 0.05). Count—number of enriched DEGs; rich factor—ratio of DEG to the total number of genes annotated in the pathway.

**Figure 4:**
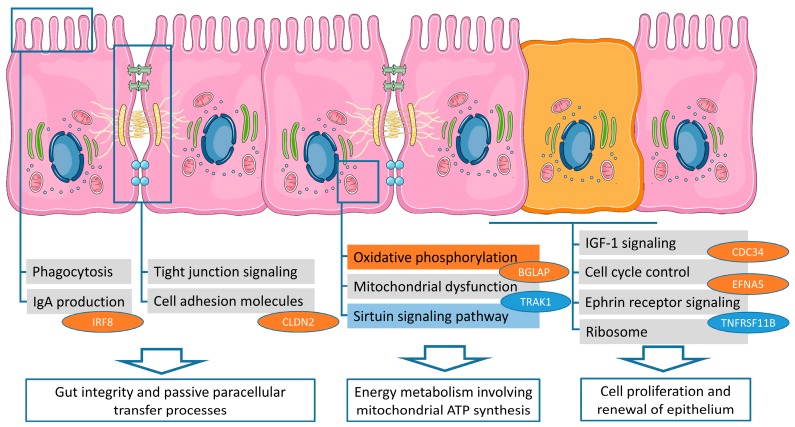
Conceptual model of the ileal epithelium of Japanese quail with divergent PU status. The model links gut integrity, energy metabolism, and enterocyte proliferation rate as potential molecular determinants for efficient PU. Pathways and vitamin D responsive hub genes indicate transcriptional up- (orange items) or down-regulation (blue items) in the high PU group compared to the low PU group. Grey boxes indicate transcriptional effects not predicted based on the Ingenuity Pathway Analysis (IPA) *z*-score.

The list of DEGs was used for gene enrichment analysis via Ingenuity Pathway Analysis (IPA; Table 1) and Kyoto Encyclopedia of Genes and Genomes (KEGG; Figure 3) to derive ileal pathways discriminating between PU groups. The corresponding results and retrieved conclusions are summarized as conceptual model to highlight the most prominent differences between Japanese quail with divergent PU (Figure 4). IPA and KEGG showed three and six significantly enriched pathways (adjusted *p*-value ≤ 0.05), revealing a substantial concordance with regards to an affected mitochondrial energy metabolism (Table 1, Figure 3). Considering the activation indices (z-scores) obtained from IPA, “oxidative phosphorylation” was predicted to be significantly activated in high compared to low PU animals (Table 1). Based on the predicted activation state (IPA *z*-score), this observation may imply PU group-specific differences in mitochondrial functions, e.g., ATP production efficiency [28]. In the intestine, mitochondria play a central role in providing energy for maintaining tissue integrity and are essential for the permanent renewal of cells in a 3 –5 day tonus [29]. These renewal processes and intestinal functions such as absorption processes consume 15%–25% of the total energy required by birds [30]. In the current study, animals with high PU appear to have a higher proliferation rate of intestinal cells, as indicated by the enrichment of the oxidative phosphorylation pathway [31]. In contrast, quail with a low PU might exhibit shifts in energy metabolism utilizing signal transduction mechanisms of the “sirtuin signaling pathway” (Table 1). Indeed, sirtuin signaling is involved in cellular processes such as glycolipid metabolism, apoptosis and in balancing mitochondrial energy metabolism and glycolysis. These findings at the pathway level indicate an important role of energy metabolism for PU in poultry, but require further conclusive physiological measurements, e.g., at the level of the mitochondria. The accumulation of DEGs involved in “IGF1 signaling”, “ephrin receptor signaling”, “Ribosome pathways”, and “cell cycle control of chromosomal replication”, indicated differences in intestinal growth and development processes between PU-divergent groups (Table 1). Therefore, the proliferation rate of intestinal cells might be part of the endogenous mechanisms of efficient PU as shown in the conceptual model in Figure 4.

Interestingly, “cell adhesion molecules”, “tight junction”, “phagocytosis and phagosomes”, and the “production of immunoglobulin A” represent specific features to gut integrity and were also among the enriched pathways in both analyses (Table 1, Figure 3). Moreover, the IGF1 system is sensitive to nutrition [32], and IGF1 is known to alter intestinal barrier permeability, at least in mammals [33]. The enriched KEGG pathway “tight junctions” represent a network of membrane proteins such as claudins and tight junction proteins (Figure 3). It is considered an essential component of the intestinal barrier by forming paracellular channels to transport selective ions and solutes. Indeed, tight junctions turned out to be highly permeable to phosphate ions along the entire gastrointestinal tract [34]. Variations in the expression of tight junction proteins might affect passive paracellular transfer processes in epithelial cells [35], which can also impact intestinal barrier integrity [36,37]. Variation of the calcium homeostasis and of vitamin D levels have been shown to regulate the expression of corresponding claudins [38,39]. It is conceivable that the high PU group manages to adapt to low P challenges engaging paracellular transfer processes as summarized in Figure 4.

### 2.4. Target Genes Attributed to the Vitamin D System

The retrieved enrichment analyses did not indicate effects on known transcellular sodium/P co-transporters or primary components affiliated with the vitamin D system. This might be attributed to the initial adaptation period on the low P diet (days 6–10), when both low and high PU animals likely induced P-related intrinsic responses to maintain overall P homeostasis. However, due to the superior control in P homeostasis we reviewed target genes attributed to the vitamin D receptor (VDR) more closely [40]. Retrieved DEGs affiliated with the VDR pathway contain transcripts such as CLDN2 (low PU < high PU; FC 2.18), TNFRSF11B (low PU > high PU; FC 3.84), BGLAP (low PU < high PU; FC 1.75), CDC34 (low PU < high PU; FC 1.33), EFNA5 (low PU < high PU; FC 1.68), IRF8 (low PU < high PU; FC 1.47), and TRAK1 (low PU > high PU; FC 1.68), which are included in Figure 4. In vitro studies have shown that tight junctions containing claudin 2 (CLDN2) proteins are responsive to the vitamin D signaling via VDR [39]. As mentioned above, the overexpression of CLDN2 in ileum of high PU quail might therefore account for improved paracellular ion fluxes, including P and calcium. A close relationship between PU and other traits such as calcium utilization is also evidenced by their positive correlations in the whole experimental quail population [4]. Interestingly, TNFRSF11B encoding osteoprotegerin (OPG) is known to be expressed in various peripheral tissues such as the intestine [41]. Moreover, it is released to the blood system [42]. OPG is discussed to be involved in the initiation of immunomodulatory responses [43], but OPG is primarily known to act as a decoy soluble receptor for the receptor activator of NF-κB ligand (RANKL). In fact, the RANK/RANKL system represents an important regulatory loop that controls maturation and activity of both osteoblasts and osteoclasts [44,45], where OPG impacts on bone resorption as a negative regulator. However, in intestine TNFRSF11B was further proposed to act as upstream regulator of enterocytes proliferation [46]. Taken together, the ileal gene expression of high PU quail might appear to be effective via VDR-responsive downstream targets to act on paracellular ion transport, mitochondrial energy metabolism, and enterocyte proliferation (Figure 4).

## 3. Materials and Methods

### 3.1. Animals and Sample Collection

In a recent study, Japanese quail have been subjected to generate a large F2-cross comprised of 920 individuals [5]. Parental lines have been selected for social reinstatement behavior [47]. The study was approved by the animal welfare commissioner of the University of Hohenheim (number S371/13TE) and conducted in accordance with animal welfare regulations. The experimental protocol has been described previously [5]. In brief, the trial consisted of 12 hatches. Animals were raised on commercial starter diets (ad libitum; days 0–5 of life) and kept in groups on floor pens. All animals were kept under equal environmental conditions. From day 6, the quail were fed a low P diet (ad libitum) and were transferred to individual metabolic cages (day 7). The experimental diet was based on corn and soybean meal. Its total P content was 4.0 g/kg dry matter, lacking both phytase and mineral P supplements. The P supply level was below current dietary recommendations to ensure that animals exploit their genetic potential in terms of PU [48]. From day 10–15, individual feed intake (FI; [g]) was recorded and the concentration of dietary P was analyzed (P_Diet_; [mg/g]). Moreover, individual excreta were collected in the respective period. The amount of excreted P was quantified (P_Excreta_; [mg]) by standard protocols as described previously [5,49]. Based on the equation proposed by Shastak and Rodehutscord [50], individual PU has been estimated: PU [%] = 100 − 100 × [(P_Excreta_)/(P_Diet_ × FI)]. The quail were slaughtered at day 15, and ileum tissue was collected [5]. Dissected ileum samples were immediately submerged in RNAlater solution (Sigma-Aldrich, Taufkirchen, Germany) and stored at −80 °C until use for total RNA extraction. For transcriptome analyses, ileal samples retrieved from discordant quail sib pairs of ten families were used (low PU: *n* = 10; high PU: *n* = 10). Each family was represented by two individuals of the same sex. Selected animals differed significantly in PU values (*p* < 0.001), i.e., high PU (mean ± SD: 79.4 ± 3.3%) or low PU (mean ± SD: 39.2 ± 11.1%), but also in calcium utilization (high PU: 70.8 ± 5.7%; low PU: 32.4 ± 11.4%; *p* < 0.001) and FCR (high PU: 1.8 ± 0.1 g/g; low PU: 2.7 ± 0.8 g/g; *p* < 0.01). The descriptive statistics (mean, minimum, maximum, standard deviation) for PU, calcium utilization, and FCR of the entire quail population have been reported previously [4].

### 3.2. RNA Preparation, Library Construction, and RNA Sequencing

Intestinal tissue samples were ground into powder in liquid nitrogen, and approximately 50 mg of the sample was homogenized in 1 ml of TRIzol Reagent (Invitrogen, Karlsruhe, Germany). Total RNA was extracted according to the manufacture instructions. Extracted RNA was additionally treated with DNaseI using RNase-Free DNase Set (Qiagen, Hilden, Germany) and purified using the RNeasy Mini Kit (Qiagen, Hilden, Germany). The RNA quantity and quality was assessed using a NanoDrop ND-2000 (Peqlab, Erlangen, Germany) and a Bioanalyzer 2100 (Agilent Technologies, Waldbronn, Germany), respectively. High quality RNA (RNA integrity number; RIN > 7.5) was used for mRNA sequencing library preparation using a TruSeq Stranded mRNA library preparation kit (Illumina, San Diego, CA, USA). Briefly, poly-A mRNA molecules were enriched using poly-T oligo attached magnetic beads followed by fragmentation and first strand cDNA synthesis with random primers. Each DNA library was barcoded with a different indexing TruSeq-adapter to enable a parallel sequencing of pooling multiple samples. The DNA libraries were quality checked using a DNA 1000 chip (Agilent Technologies, Waldbronn, Germany) and quantified for molar concentration using the Qubit dsDNA HS assay kit (Invitrogen, Darmstadt, Germany) prior to sequencing on the Illumina HiSeq 2500 sequencing platform. Sequencing reads were generated for a paired-end of 2 × 101 bp. The data of mRNA sequencing have been assigned with the accession number E-MTAB-8587 in the ArrayExpress database at EMBL-EBI (www.ebi.ac.uk/arrayexpress).

### 3.3. Data Analyses

Adaptor sequences and reads which did not meet quality criteria were initially removed from raw data. The remaining reads were aligned to the reference genome of *Coturnix japonica* (genome build 2.0; GCF_001577835.1; https://www.ncbi.nlm.nih.gov/genome), using STAR 2.6.1b [51]. Default settings of the STAR command were used, whereby the “sjbdOverhang” option was adapted to the length of sequencing reads and set to 100. Subsequently, the quantification of aligned reads was performed using the software package StringTie (version 1.3.4), and raw transcript counts were obtained by the corresponding python script [52]. The initial dataset contained 16,037 gene entries.

The R package DESeq2 (version 1.22.2) [53] was used for the differential gene expression analysis. In an initial filtering step, genes having zero counts in at least 50% of the samples were excluded. The design of the statistical model considered the predictors “family” and “PU group”. The contrasts between PU groups were obtained using the Wald test. Finally, false discovery rates (q-values) were calculated using the R package q-value [54,55], and genes were reported to be differentially expressed if q ≤ 0.05. Regularized-log-transformed expression values of these differentially expressed genes were used for principle component analysis (PCA) based on singular value decomposition on the expression matrix. The first two principle components were illustrated with the gplots R package (v3.0.1.1).

For functional analysis, gene identifiers were converted to human orthologue Ensembl identifiers if available or quail gene symbols using g:Profiler (http://biit.cs.ut.ee/gprofiler). Gene ontology analysis was performed using protein analysis through evolutionary relationships (PANTHER; version 14) [56] against the human knowledge base. This further comprised overrepresentation analysis of GO terms against the human reference list (including 20,996 genes) using Fisher’s exact test of independence with false discovery rate correction (q-value) and a q-value cut-off of 0.05. 

Enrichment analysis of genes into certain pathways was done by KOBAS 3.0 [57] based on the Kyoto Encyclopedia of Genes and Genomes (KEGG) database. Furthermore, the Ingenuity Pathway Analysis (IPA) software based on the manually curated Ingenuity Knowledge Base was applied. For KOBAS and IPA, Fisher’s exact test were performed and pathways were considered to be significantly enriched at a Benjamini–Hochberg adjusted *p*-value ≤ 0.05. A conceptual model was created using images taken from Sevier Medical Art (https://smart.servier.com).

## 4. Conclusions

Ileum transcriptome analysis of quail siblings discordant for high- and low-PU under low dietary P condition reveals molecular pathways related to intermediate metabolism and mechanisms of resource allocation (see also Figure 4). Animals exhibiting high PU revealed significant altered signaling pathways involved in mitochondrial energy metabolism, accelerated cell proliferation of enterocytes and gut integrity. As PU does not appear to be fully independent from traits like calcium utilization and feed efficiency, further advances in developing indicators for the efficient use of both macro- and micronutrients are required. Turn-over and proliferation rates of intestinal cells could be an important issue in further research on PU and investigations should be extended to laying hens to establish consistent mechanisms of PU in poultry.

## Figures and Tables

**Table 1:** Significantly enriched canonical pathways deduced from genes found to be differentially expressed between low and high PU groups.

Canonical Pathways	*P*-Value ^1^	z-Score ^2^	Molecules
Mitochondrial Dysfunction *	<0.001		ATP5F1D, ATP5MG, CASP9, COX5B, COX6A1, COX7B, MAOB, MT-ND6, NDUFA11, NDUFA13, NDUFB1, NDUFB6, NDUFB9, NDUFV1, SDHC, TRAK1, UQCR11
Oxidative Phosphorylation *	<0.001	3.6	ATP5F1D, ATP5MG, COX5B, COX6A1, COX7B, NDUFA1, NDUFA13, NDUFB1, NDUFB6, NDUFB9, NDUFV1, SDHC, UQCR11
Sirtuin Signaling Pathway *	<0.001	−2.3	ATP5F1D, GABARAPL1, GLS, HIST1H1T, MT-ND6, NDUFA11, NDUFA13, NDUFB1, NDUFB6, NDUFB9, NDUFV1, NFKB2, OGG1, PCK1, SDHC, TOMM6, TP53BP1, XPC
Fcγ Receptor-mediated Phagocytosis in Macrophages and Monocytes	0.003	1.4	ARPC3, ARPC5L, FGR, HMOX1, NCF1, NCK2, PIK3CG, RPS6KB2
Cell Cycle Control of Chromosomal Replication	0.003		CDK10, CDK13, CDK4, POLE, RPA3, TOP2A
Tight Junction Signaling	0.003		AFDN, CDK4, CLDN2, CLDN23, CLDN4, MYH6, NFKB2, PATJ, PPM1J, TJP1, TNFRSF11B
IGF-1 Signaling	0.005	−1.3	CASP9, IGF1R, IGFBP2, IGFBP7, PIK3CG, RASD1, RPS6KB2, SOCS3
Ephrin Receptor Signaling	0.006	−0.7	ARPC3, ARPC5L, ATF2, EFNA5, EPHB6, GNAL, GNG13, NCK2, PIK3CG, RASD1, SORBS1
Sertoli Cell-Sertoli Cell Junction Signaling	0.007		AFDN, ATF2, CLDN2, CLDN23, CLDN4, MPP6, PRKG1, RASD1, SORBS1, SPTBN1, TJP1

* Benjamini–Hochberg adjusted *p*-value ≤ 0.05; ^1^ displayed *p*-value ≤ 0.01; ^2^ z-score: Pathways with a z-score > 2 and z-score < −2 were considered as significantly activated and inhibited in high PU quail, respectively.

## Supplementary Materials

Supplementary Table S1. List of differentially expressed genes (DEGs) derived from ileal tissue of quail discordant for phosphorus utilization (xlsx). Associated statistics (*p*-value, q-value, fold change) are provided. Supplementary materials can be found at https://www.mdpi.com/1422-0067/21/8/2762/s1.

## Abbreviations

CLDN2	Claudin 2
FI	Feed intake
GO	Gene ontology
InsP6	Inositol hexakisphosphate
IPA	Ingenuity pathway analyses
KEGG	Kyoto Encyclopedia of Genes and Genomes
OPG	Osteoprotegerin
P	Phosphorus
PU	Phosphorus utilization
RANK	Receptor Activator of NF-κB
RANKL	Receptor Activator of NF-κB Ligand
VDR	Vitamin D receptor
